# To assess the effective and safety of compound glutamine entersoluble capsules in irritable bowel syndrome: A protocol for systematic review and meta-analysis

**DOI:** 10.1097/MD.0000000000025098

**Published:** 2021-03-12

**Authors:** Yong Zhang, Ru Liu, Jin Wang, Shuguang Yan, Zhiqing Guo

**Affiliations:** a Department of Gastroenterology, Sichuan Second Chinese Medicine Hospital; b College of Computer Science, Chengdu University, Chengdu, Sichuan; c Shaanxi University of Chinese Medicine, Xianyang, Shaanxi; d Pharmacy Department, Hospital of Chengdu University of Traditional Chinese Medicine, Chengdu, Sichuan, China.

**Keywords:** compound glutamine entersoluble capsules, irritable bowel syndrome, protocol, systematic review and meta-analysis

## Abstract

**Background:**

Irritable bowel syndrome (IBS) is one the common medical condition of functional GI disorder (FGD) characterized by bowel-related symptoms without other organic gastrointestinal (GI) disease. Compound Glutamine Entersoluble Capsules(CGEC),a compound preparation in which each capsule contains 120 mg L-glutamine, 50 mg ginseng, 50 mg licorice, 50 mg Atractylodes macrocephala and 50 mg Poria cocos, have been reported the efficacy of CGEC for patients with IBS in improving the clinical symptoms and quality of patients’ life. However, there is no a systematic review related to CGEC for IBS to this day. In this study, we will systematically evaluate the effectiveness and safety of CGEC in the treatment of IBS-D with a meta-analysis method, so as to provide a solid evidence for clinical practice.

**Methods:**

In this study, a literature search was performed by using the Chinese and English databases, which include PubMed, Embase, MEDLINE, Cochrane Library Central Register of Controlled Trials, China National Knowledge Infrastructure (CNKI) database, Wanfang Data Knowledge Service Platform, the VIP information resource integration service platform (cqvip), China Biology Medicine Disc (Sino Med),and the Chinese Clinical Trial Registry (ChiCTR), to find the related literature of CGEC in the treatment of IBS published from the inception date of each predefined database upto January 2021. The evaluation of the risk of bias for eligible studies will be performed by two investigators. Data synthesis will be performed by RevMan 5.4 software. Heterogeneity between studies can be assessed by a heterogeneity X2 test. The degree of heterogeneity among multiple included studies can be measured by I^2^. The stability of systematic review or meta-analysis outcomes will be evaluated by Sensitivity analysis. Reporting bias will be evaluated by funnel plot. Finally, The Grading of Recommendations Assessment, Development and Evaluation (GRADE) will be used to assess the quality of evidence obtained.

**Results:**

The results of this study will be published in a peer-reviewed journal.

**Conclusion:**

Whether it is the effectiveness and safety of CGEC in the treatment of IBS will be judged in the result of this systematic review.

## 1 Introduction

Irritable bowel syndrome (IBS) is one the common medical condition of functional gastrointestinal (GI) disorder characterized by bowel-related symptoms without other organic gastrointestinal (GI) disease.^[1]^ The worldwide prevalence of IBS was 11.2% (95% confidence interval, 9.8%–12.8%) on the basis of a meta-analysis of 80 studies involving 260,960 subjects^[2]^ and the prevalence in China was 6.5% (3). The prevalence rate of women with IBS is higher than for men.^[2,3]^ The increasing costs of medical care for people with IBS leads to a negative economic impact.^[4]^ Studies also showed that the incidence rate of anxiety or depression increased 3 times for patients with IBS than for general populations^[5]^ and children with IBS had a 4 times higher risk of having celiac disease than healthy subjects,^[6]^ which leads to a lower quality of life. The cause is thought to be a multi-dimensional disorder with an interaction between gut microbial dysbiosis, GI low grade inflammation, GI infection, increased gut permeability, food intolerance, GI dysmotility, visceral hypersensitivity, altered gut-brain interaction, genetic, and psychosocial factors.^[7]^ The diagnosis for IBS is based on the Rome IV criteria, which classified as IBS with predominant constipation, IBS with predominant diarrhea, IBS with mixed bowel habits, and IBS unclassified according to disease symptoms.^[8]^ Because of the underlying cause unknown, the treatment goals of IBS are to improve patients’ symptoms and quality of life.^[9]^ The leading symptoms of IBS include abdominal pain, bloating, urgency, straining, feeling of incomplete defecation, or discomfort either improved or aggravated by passing stool or flatus.^[7]^ Nonpharmacological treatments of IBS include lifestyle modifications (including exercise, stress reduction, and attention to impaired sleep), dietary fiber supplementation, dietary restriction of gluten, and psychological and behavioral treatments.^[10]^ The dug therapeutic options of IBS based on symptom type include opioid agonists, bile salt sequestrants, probiotics, antibiotics, 5-HT3 antagonists, chloride channel activators, guanylate cyclase C agonists, smooth muscle antispasmodics, peppermint oil, tricyclic antidepressants, selective serotonin reuptake inhibitors, chloride channel activators, and guanylate cyclase C agonists. However, long-term drug use can cause many adverse events,^[10]^ which has limited its clinical application.

Compound glutamine entersoluble capsules (CGEC), a compound preparation in which each capsule contains 120 mg L-glutamine, 50 mg ginseng, 50 mg licorice, 50 mg Atractylodes macrocephala, and 50 mg Poria cocos, is developed by Diao Group Chengdu Pharmaceutical Co., Ltd (26 Chuangye Road, Gaoxin Avenue, Gaoxin District, Chengdu City, Sichuan Province, China). Experiments have shown that CGEC has a variety of pharmacological effects, such as improving the absorption, secretion and movement function of the intestinal tract,^[11]^ enhancing the intestinal mucosal barrier function,^[12]^ preventing or reducing the intestinal bacteria and toxins into the blood,^[13]^ and promoting the recovery and functional reconstruction of the damaged intestinal mucosa.^[14]^ It is used for acute and chronic intestinal diseases caused by various reasons, such as intestinal dysfunction^[15]^ and noninfectious diarrhea,^[16]^ and approved by the China Food and Drug Administration for the treatment of IBS (approved No. H51023598). Recently, more and more clinical studies have reported the efficacy of CGEC for patients with IBS in improving the clinical symptoms and quality of patients’ life.^[17,18]^ The possible mechanism of CGEC in treatment of IBS is in connection with improving intestinal mucosal barrier function,^[19]^ regulating GI hormone secretion,^[20]^ and correcting gut microbial dysbiosis.^[21]^ However, there is no a systematic review related to CGEC for IBS to this day. In this study, we will systematically evaluate the effectiveness and safety of CGEC in the treatment of IBS with a meta-analysis method, so as to provide a solid evidence for clinical practice.

## 2 Methods and analysis

### 2.1 Study registration

This protocol report has been registered at Open Science Framework (OSF, https://osf.io/), an open-source project management tool that supports researchers throughout their entire project lifecycle. The registration DOI of the report is 10.17605/OSF.IO/TDZYK. The protocol report development process is carried out in compliance with the preferred reporting items for systematic reviews and meta-analyses protocols statement guidelines.^[22]^

### 2.2 Inclusion and exclusion criteria

#### 2.2.1 Types of studies

The type of study design of this protocol will be limited to randomized controlled trials (RCTs), excluding self-controlled studies, non-RCTs, randomized crossover studies, quasi-randomized trials, unequal randomized controlled trials, cluster randomized controlled trials, animal mechanism studies, and case reports.

The articles on IBS in English and Chinese will be included.

#### 2.2.2 Types of participants

All eligible participants are diagnosed with IBS by the Rome III criteria or the Rome IV criteria and careful exclusion of other organic GI disease, taking no account of age, gender, region, education, economic status, and other factors.

#### 2.2.3 Interventions/comparators

We included those studies in which interventions involved CGEC alone or combined with other routine pharmacotherapy, and the control group includes placebo control, no treatment, and conventional treatments, such as loperamide, 5-ht3 antagonists, and Chinese herbal compound. The method of administration can be oral, and the minimum treatment duration above studies is 7 days.

#### 2.2.4 Outcomes

The primary outcomes of this review will focus on a composite of relief in both abdominal pain and stool consistency, and the secondary outcomes include the improvement of the other clinical symptoms, such as bloating, urgency, straining, feeling of incomplete defecation, or discomfort either improved or aggravated by passing stool or flatus. Any adverse events will also be included in the work.

### 2.3 Study search

In this study, a literature search was performed by using the Chinese and English databases, which include PubMed, Embase, MEDLINE, Cochrane Library Central Register of Controlled Trials, China National Knowledge Infrastructure database, Wanfang Data Knowledge Service Platform, the VIP information resource integration service platform, China Biology Medicine Disc, and the Chinese Clinical Trial Registry, to find the related literature of CGEC in the treatment of IBS published from the inception date of each predefined database upto January 2021. In addition, Google scholar, Bing scholar, and Baidu scholar will be used to find unpublished trials or supplementary data for potentially eligible studies. The above literature in English and Chinese will be limited. According to the Cochrane Handbook guidelines,^[23]^ Search strategy will be performed. The search strategy was as follows:

1#: Search: ((((((((Irritable Bowel Syndromes[Title/Abstract]) OR (Syndrome, Irritable Bowel[Title/Abstract])) OR (Syndromes, Irritable Bowel[Title/Abstract])) OR (Colon, Irritable[Title/Abstract])) OR (Irritable Colon[Title/Abstract])) OR (Colitis, Mucous[Title/Abstract])) OR (Colitides, Mucous[Title/Abstract])) OR (Mucous Colitides[Title/Abstract])) OR (Mucous Colitis[Title/Abstract])

2#: Search: ((((Compound Glutamine Entersoluble Capsules[Title/Abstract]) OR (Compound Glutamine Entersoluble tablet[Title/Abstract])) OR (Compound Glutamine Entersoluble[Title/Abstract])) OR (Gu Shen Chang An[Title/Abstract])) OR (Gu Shen Chang An Capsules[Title/Abstract])

3#:Search:(((((((((randomized controlled trial[Title/Abstract]) OR RCT[Title/Abstract]) OR random[Title/Abstract]) OR randomly[Title/Abstract]) OR random allocation[Title/Abstract]) OR allocation[Title/Abstract]) OR randomized control trial[Title/Abstract]) OR controlled clinical trial[Title/Abstract]) OR clinical trial[Title/Abstract]) OR clinical study[Title/Abstract]

#1 and #2 and #3

### 2.4 Study selection

**Figure 1:**
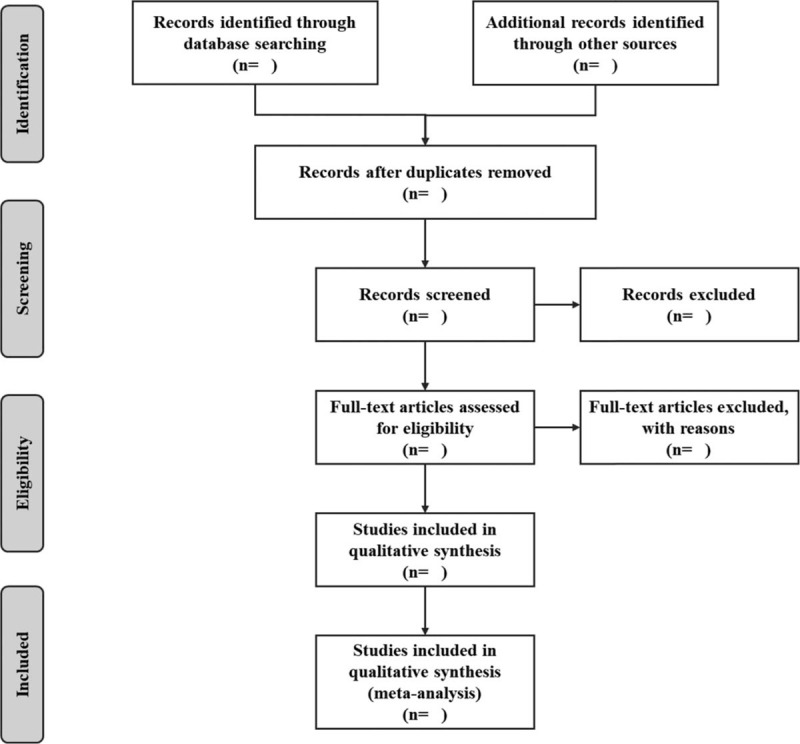
Flow chart of study selection.

Two professional trained investigators (Yong Zhang and Zhiqing Guo) will independently conduct the study search and use EndNote X9.0 (Stanford, Connecticut, https://endnote.com) to establish a citations database. Then the 2 researches will again independently read the titles and abstracts identified by the search for eligibility. According to the predefined criteria, eligible papers will be obtained and further evaluated by reading the full text in detail, and the excluded literature also will be recorded and explained. Any disagreements on the literature will be resolved by discussion. The procedure of study selection is shown in a preferred reporting items for systematic reviews and meta-analyses flow chart (Fig. 1).

### 2.5 Data extraction and management

Data will be extracted independently by 2 authors to a Microsoft Excel, again with any disagreements resolved by discussion. The following data include the first authors, year(s) conducted, country and geographic region, publication type, treatment duration, types of studies, gender, age, interventions in experimental group and control group, ample size in each group, outcome indicators, and adverse events. Where missing or unclear data are found and multiple study reports from a single study appear to exist, we will contact study authors to clarify this issue.

### 2.6 Assessment of risk of bias in included studies

The evaluation of the risk of bias for eligible studies will be performed by 2 investigators, according to the Cochrane Collaboration's bias risk assessment tool from the following aspects: random sequence generation (selection bias), allocation concealment (selection bias), blinding of participants and personnel (performance bias), blinding of outcome assessment (detection bias), incomplete outcome data (attrition bias), selective reporting (reporting bias), and other bias. The assessment of risk of bias in included studies will be divided into “Low risk,” “High risk,” or “Unclear risk.” Disagreements between investigators will be resolved by consensus.

### 2.7 Data synthesis

Data synthesis, a combination of the results of several similar studies into a single effect size, will be performed by RevMan 5.4 software. The odd ratio, relative risk, or risk difference can be selected for dichotomous variables and the mean difference or standard mean difference should be selected for continuous variables. The confidence intervals for the above variables will be set to 95%.

### 2.8 Assessment of heterogeneity

The heterogeneity test, also known as homogeneity test, is a method used to test whether the statistics of multiple similar studies have heterogeneity. Heterogeneity between studies can be assessed by a heterogeneity *χ*^2^ test. If *P*-value > .10, the fixed-effects model will be used to synthesize the data. If *P*-value ≤ .10, the causes of heterogeneity will be analyzed by the subgroup analysis from the following aspects: the design scheme, measurement scheme, dosage, medication method, age, gender, course of disease, and other factors. The degree of heterogeneity among multiple included studies can be measured by *I*^2^. As long as it is less than 50%, heterogeneity is acceptable.

### 2.9 Sensitivity analysis

The stability of systematic review or meta-analysis outcomes will be evaluated by Sensitivity analysis, which includes the following aspects: Changing the inclusion criteria, research objects, interventions or endpoint indicators of the study type, reanalysis of data using different statistical methods, and analyzing again the data after reasonably evaluating the missing date. If there will be no essential changing outcomes of systematic review or meta-analysis, the reliability of the analysis results will be greatly increasing. On the contrary, the study results should be interpreted with caution.

### 2.10 Assessment of reporting biases

Reporting bias refers to the systematic differences between the results reported in the article and those measured but not reported, which includes the following aspects: publication bias, time lag bias, duplication publication bias, location bias, citation bias, language bias, and outcome reporting bias. Reporting bias will be evaluated by funnel plot regarded as a general method to show the small-study-effects. If the funnel plot is asymmetric, it indicates that the reporting bias exists, and the more obvious the asymmetry is, the greater the degree of bias is.

### 2.11 Grading the quality of evidence

The grading of recommendations assessment, development, and evaluation, a widely used tool in evaluating the quality of assessment,^[24]^ will be used to assess the quality of evidence obtained. According to the methodology of study design, the quality of evidence is divided into 4 grades: “high,” “moderate,” “low,” and “very low.”

### 2.12 Patient and public involvement

There are no patient and public involving in this study.

### 2.13 Ethics and dissemination

It is not necessary for the ethical approval in this study, because the data extracted does not involve individual patients. The purpose of the study is to provide solid evidence for clinical practice, and the results of the study will be published in a peer-reviewed journal.

## 3 Discussion

IBS is one of the most common functional bowel disorders, and there is a substantial impact on quality of life for patients with active symptoms.^[25]^ Because of the growing costs of medical care for people with IBS,^[26]^ a negative economic impact also has been led. Although the efficacy of CGEC for IBS in recent clinical studies has been reported, it is not recommended for treatment of IBS by expert consensus. Therefore, a systematic review and meta-analysis of the relevant studies is urgently needed to conduct, which can provide solid evidence for the clinical application of CGEC for IBS.

### 3.1 Amendments

If the protocol is needed to modify in the process of research, the information will be updated in the final report.

## Author contributions

**Conceptualization:** Yong Zhang, Zhiqing Guo.

**Data curation:** Yong Zhang, Zhiqing Guo.

**Formal analysis:** Yong Zhang, Ru Liu, Jin Wang.

**Funding acquisition:** Shuguang Yan.

**Investigation:** Yong Zhang, Zhiqing Guo.

**Methodology:** Yong Zhang, Zhiqing Guo.

**Project administration:** Yong Zhang, Zhiqing Guo.

**Resources:** Yong Zhang, Zhiqing Guo.

**Software:** Yong Zhang, Zhiqing Guo.

**Supervision:** Ru Liu, Jin Wang.

**Writing – original draft:** Yong Zhang.

**Writing – review & editing:** Yong Zhang, Zhiqing Guo.

## References

[1] Defrees DN Bailey J. Irritable bowel syndrome: epidemiology, pathophysiology, diagnosis, and treatment. Prim Care 2017;44:655–71. 29132527 10.1016/j.pop.2017.07.009

[2] Lovell RM Ford AC. Global prevalence of and risk factors for irritable bowel syndrome: a meta-analysis. Clin Gastroenterol Hepatol 2012;10:712–21.e4. 22426087 10.1016/j.cgh.2012.02.029

[3] Zhang L Duan L Liu Y. A meta-analysis of the prevalence and risk factors of irritable bowel syndrome in Chinese community. Zhonghua Nei Ke Za Zhi 2014;53:969–75. 25623565

[4] Hauser W Marschall U Layer P. The prevalence, comorbidity, management and costs of irritable bowel syndrome. Dtsch Arztebl Int 2019;116:463–70. 31431234 10.3238/arztebl.2019.0463 PMC6718888

[5] Zamani M Alizadeh-Tabari S Zamani V. Systematic review with meta-analysis: the prevalence of anxiety and depression in patients with irritable bowel syndrome. Aliment Pharmacol Ther 2019;50:132–43. 31157418 10.1111/apt.15325

[6] Cristofori F Fontana C Magista A. Increased prevalence of celiac disease among pediatric patients with irritable bowel syndrome: a 6-year prospective cohort study. JAMA Pediatr 2014;168:555–60. 24756157 10.1001/jamapediatrics.2013.4984

[7] Gwee KA Gonlachanvit S Ghoshal UC. Second Asian consensus on irritable bowel syndrome. J Neurogastroenterol Motil 2019;25:343–62. 31327218 10.5056/jnm19041 PMC6657923

[8] Drossman DA. Functional gastrointestinal disorders: history, pathophysiology, clinical features and Rome IV. Gastroenterology 2016;150:1262–79. 10.1053/j.gastro.2016.02.032 27144617

[9] Cooperative Group of Gastrointestinal Functional Diseases GB, Chinese Medical Association GDG, Digestive Society of Chinese Medical Association. Expert consensus on irritable bowel syndrome in China in 2020. Chin J Dig 2020;40:803–18.

[10] Mearin F Lacy BE Chang L. Bowel disorders. Gastroenterology 2016;150:1393–407. 10.1053/j.gastro.2016.02.031 27144627

[11] Qi Z. Compound glutamine with probiotics in prevention of fluorouracil chemotherapy associated diarrhea. Sichuan Med J 2016;37:47–9.

[12] Yandong X Jingyu Z Qingling F. Effects of Shenling Baizhu powder combined with compound glutamine on intestinal mucosal barrier function and levels of 5-HT, IFN-γ and IL-8 in patients with irritable bowel syndrome. Prog Mod Biomed 2019;19:4269–72.

[13] Hao H Mingkai C Lingwen D. Effect of probiotics combined with compound glutamine on the prevention of spontaneous bacterial peritonitis in patients with decompensated cirrhosis. J Med Res 2014;43:25–8.

[14] Hongtao S Zhilong D Enreng L. Study of protective effects of GuShenChangAn on intestines in rabbit during ischemia reperfusion. Chongqing Med J 2002;31:96–7.

[15] Liping Z Wu W. Effects of bifidobacterium combined with compound glutamine enteric-coated capsules on intestinal mucosal barrier and inflammatory cytokines in patients with gastrointestinal disorders. Sichuan J Physiol Sci 2018;40:102–4.

[16] Biwu J Yixi L Guizhen L. Golden Bifid combined with compound glutamine in the treatment of 58 cases of chronic non infectious diarrhea. Chin J Integrated Tradit West Med Dig 2006;14:336–7.

[17] Zhongliang D Yukun J Yinhua Z. Clinical observation on Gushen Changan in treating 38 cases of diarrhea predominant irritable bowel syndrome. Clin Med China 2002;18:591.

[18] Hongxia C. Treatment of 60 cases of irritable bowel syndrome with Gushen Changan capsule. Shaanxi J Tradit Chin Med 2007;28:1214.

[19] Meizhu Y Manru S Ying C. Effect of compound glutamine on intestinal mucosal barrier function in patients with diarrhea predominant irritable bowel syndrome. J Chin Physician 2018;20:745–6.

[20] Guo S. Effect of compound glutamine entersoluble capsules on gastrointestinal hormone levels in patients with irritable bowel syndrome. J North Pharm 2017;14:54–5.

[21] Jun C Zebo J Yongxi W. Therapeutic effect evaluation of combination glutamine capsule combined with bifidobaeterium tetravaccine tablets in the treatment of intestinal dysbacteriosis-induced diarrhea. J Hunan Normal Univ (Med Sci) 2016;13:113–5.

[22] Moher D Shamseer L Clarke M. Preferred reporting items for systematic review and meta-analysis protocols (PRISMA-P) 2015 statement. Syst Rev 2015;4:1–9. 25554246 10.1186/2046-4053-4-1 PMC4320440

[23] Shuster JJ Higgins JPT Green S. Cochrane handbook for systematic reviews for interventions, Version 5.1. 0, published 3/2011. Res Synth Methods 2011;2:126–30

[24] Atkins D Best D Briss PA. Grading quality of evidence and strength of recommendations. BMJ 2004;328:1490–4. 15205295 10.1136/bmj.328.7454.1490 PMC428525

[25] Wang YT Lim HY Tai D. The impact of irritable bowel syndrome on health-related quality of life: a Singapore perspective. BMC Gastroenterol 2012;12:1–5. 22873839 10.1186/1471-230X-12-104 PMC3436771

[26] Akehurst RL Brazier JE Mathers N. Health-related quality of life and cost impact of irritable bowel syndrome in a UK primary care setting. Pharmacoeconomics 2002;20:455–62. 12093301 10.2165/00019053-200220070-00003

